# A century of canopy kelp persistence and recovery in the Gulf of Alaska

**DOI:** 10.1093/aob/mcad149

**Published:** 2023-10-13

**Authors:** Jordan A Hollarsmith, Juliana C Cornett, Emily Evenson, Alex Tugaw

**Affiliations:** NOAA, National Marine Fisheries Service, Alaska Fisheries Science Center, 17109 Point Lena Loop Road, Juneau, AK 99801, USA; NOAA, National Marine Fisheries Service, Alaska Fisheries Science Center, 17109 Point Lena Loop Road, Juneau, AK 99801, USA; Alaska Sea Grant, University of Alaska Fairbanks, 218 O’Neill Building, PO Box 755040, Fairbanks, AK 99775, USA; Washington State University, 1815 Wilson Road, Pullman, WA 99163, USA; Cooperative Institute for Climate, Ocean, & Ecosystem Studies, University of Washington, 3737 Brooklyn Avenue NE, Seattle, WA 98105, USA and; University of Alaska Southeast, 11066 Auke Lake Way, Juneau, AK 99801, USA

**Keywords:** Sea otter, bull kelp, *Nereocystis luetkeana*, giant kelp, *Macrocystis pyrifera*, dragon kelp, *Eularia fistulosa*, Gulf of Alaska, ShoreZone, volcanic eruption, potash, Kelp Watch

## Abstract

**Background and Aims:**

Coastal Alaska contains vast kelp habitat that supports diverse marine and human communities. Over the past century, the North Pacific Ocean has undergone oceanographic and ecological regime shifts that have the potential to influence the structure and function of kelp ecosystems strongly. However, the remoteness and complexity of the glacially carved region precludes the regular monitoring efforts that would be necessary to detect such changes.

**Methods:**

To begin to fill this critical knowledge gap, we drew upon historical and modern surveys to analyse the change in spatial coverage and species composition of canopy kelp between two time points (1913 and the early 2000s to 2010s). We also incorporated decadal surveys on sea otter range expansion following complete extirpation and reintroduction to assess the influence of sea otter recovery on the spatial extent of canopy kelp.

**Key Results:**

We found increases in the spatial extent of canopy kelp throughout the Gulf of Alaska where there was coverage from both surveys. Kelp in Southcentral Alaska showed extensive recovery after the catastrophic Novarupta volcano. Kelp in Southeast Alaska showed persistence and spatial increase that closely matched increases in the range of sea otters. Observations of thermally tolerant kelp species increased more than observations of cold-adapted species between the two surveys.

**Conclusions:**

Contrary to trends observed at lower latitudes, the kelp forests that ring the Gulf of Alaska have been remarkably stable and even increased in the past century, despite oceanographic and ecosystem changes. To improve monitoring, we propose identification of sentinel kelp beds for regular monitoring to detect changes to these iconic and foundational canopy kelp species more readily.

## INTRODUCTION

Kelp, large brown macroalgae in the order Laminariales, are culturally and economically important species and are foundational to diverse ecosystems in nearshore temperate zones globally (Teagle *et al.*, 2017). Kelp themselves are an important source of food, a navigational tool and a source of textiles for human societies in modern and ancient times (e.g. Erlandson *et al.*, 2007; Thurstan *et al.*, 2018; Naar, 2020). The habitat created by kelp is valued in the billions of dollars owing to the ecosystem services for fisheries, nutrient cycling and carbon removal, and kelp species are increasingly cultivated for aquaculture (Eger *et al.*, 2023). However, as the climate warms and humans fish down the food web, kelp ecosystems worldwide are diminishing (Pauly and Palomares, 2005; Krumhansl *et al.*, 2016). In Western Australia and Northern California, for example, marine heatwaves decimated kelp ecosystems, with lasting impacts even after the heatwave event passed (McPherson *et al.*, 2021; Wernberg, 2021). In both cases, change was rapidly detectable owing to regular monitoring efforts.

Kelp habitat in the North Pacific is extensive, diverse and evolutionarily ancient, yet a lack of regular monitoring efforts has precluded our ability to detect change readily, despite major oceanographic and ecological shifts over the past century (Esslinger and Bodkin, 2009; Bolton, 2010; Marshall *et al.*, 2019; Danielson *et al.*, 2022). The most notable of these changes are an oceanographic shift in the 1980s towards warmer sea surface temperatures in the Gulf of Alaska (Marshall *et al.*, 2019; Danielson *et al.*, 2022) and range expansions of sea otters (*Enhydra lutis*) following near-total extirpation owing to the Russian fur trade in Southeast Alaskan waters in the 19^th^ century (Esslinger and Bodkin, 2009). Sea otters are prolific consumers of benthic invertebrates, including species that consume kelp, such as sea urchins. In general, the presence of sea otters results in a reduction of benthic herbivores and a resulting increase in kelp (e.g. Breen *et al.*, 1982; Estes and Duggins, 1995), although this positive relationship between otters and kelp can be mediated by confounding oceanographic and demographic factors (e.g. Watanabe and Harrold, 1991; Konar, 2000). Therefore, although the human population density in the North Pacific, specifically the Gulf of Alaska, remains low, regime shifts driven by anthropogenic and oceanographic forcing might still be altering kelp ecosystems significantly (Parnell *et al.*, 2010; Robinson *et al.*, 2020).

**Fig. 1.**
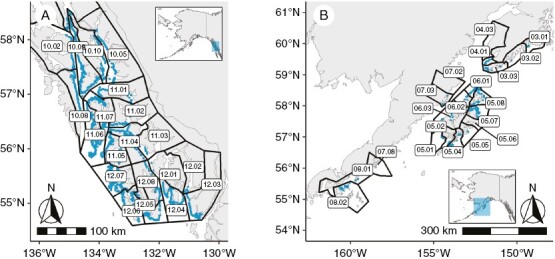
The area of study, with ShoreZone units outlined in blue and ShoreZone Areas indicated as black polygons in Southeast Alaska (A) and Southcentral Alaska (B). The number before the decimal point in the polygon labels is the ShoreZone region, and the number after the decimal point is the ShoreZone area within that region.

To address this major knowledge gap in the spatial and temporal dynamics of Gulf of Alaska canopy kelp ecosystems, we drew upon the only two wide-scale kelp surveys ever conducted in the region to assess the change in the linear extent and species composition of canopy kelp beds in Southeast and Southcentral Alaska (Fig. 1). The first datasets were maps of canopy kelp created by expeditions in the early 20th century to investigate domestic sources of potash salts, an important component of agricultural fertilizer (Cameron, 1915; Cameron hereafter). The second were the ShoreZone surveys that mapped 94% (>120 000 km) of the nearshore habitat in Alaska from 2001 to 2016 (ShoreZone, 2022; ShoreZone hereafter). Both datasets included information on the linear extent and density of the three kelp species in the North Pacific that form floating canopies: dragon kelp (*Eularia fistulosa*), bull kelp (*Nereocosytis luetkeana*) and giant kelp (*Macrocystis pyrifera*). Historical maps of canopy kelp extent have been used to great effect in Washington and British Columbia to extend our knowledge of kelp ecosystems over time (Costa *et al.*, 2020; Berry *et al.*, 2021). This is the first time that the Cameron Alaska expedition dataset has been digitized and georeferenced. We also incorporated surveys of sea otter range extent in Southeast Alaska from 1968 to 2003 to assess the influence of trophic recovery on kelp ecosystems (Esslinger and Bodkin, 2009). By combining these diverse data sources, we provide the first spatially and temporally extensive analysis of Alaskan canopy kelp ecosystems, and we have found evidence for increases in kelp extent after trophic and geological disturbance, and overall stability of kelp ecosystems despite oceanographic changes.

## MATERIALS AND METHODS

### Species of focus

This study focuses on the three floating kelp species found in Alaska. Dragon kelp (*E. fistulosa*), in the family Alariaceae, is an annual subpolar species with a range extending from Southeast Alaska to the Aleutian Islands and to Japan and Russia. Dragon kelp grows in semi-protected to exposed habitats (Lindeberg and Lindstrom, 2016). Bull kelp (*N. luetkeana*), in the family Laminariaceae, is an annual temperate species found from Southern California to the Aleutian Islands. It is most commonly found in semi-exposed or high-current areas (Lindeberg and Lindstrom, 2016). Giant kelp (*M. pyrifera*), also in the family Laminariaceae, is the only perennial canopy species in Alaska and is found in semi-exposed habitats from Baja California, Mexico, to the Kodiak Archipelago in Alaska (Lindeberg and Lindstrom, 2016).

### Overview of datasets

The Cameron surveys were conducted visually from vessels driven 20–400 yards (18–365 m) away from the kelp being mapped. The width of kelp beds was estimated visually using cues from the shoreline. In Southeast Alaska, surveys took place from May to September 1913, and in Southcentral Alaska they took place from July to August 1913. The survey report did not state expressly where the expeditions did or did not visit (Cameron, 1915).

The ShoreZone surveys were conducted by capturing the shoreline using high-resolution video and photographs from helicopter or fixed wing aircraft 100 m offshore and at 100–300 m elevation. Flights took place during spring and summer daylight tides lower than 0 m (mean low water). Based on the collected imagery, the shoreline was segmentized into relatively homogeneous units based on biological and geomorphic features (Cook *et al.*, 2017). The wave exposure classification within each unit was defined by ShoreZone as the effective fetch range, which is the distance wind can travel across open water. Owing to the extensive coastline to be mapped and variation in available funding, surveys took place over several years. Southeast Alaska was mapped between 2004 and 2013. Most of Southcentral Alaska that was included in our analyses was mapped from 2001 to 2003, with some shoreline mapped from 2008 to 2016. Each location was mapped only once over the course of these years (Cook *et al.*, 2017; Fig. 1).

Surveys of sea otter range were conducted by the United States Fish and Wildlife Service (Esslinger and Bodkin, 2009). Otters were counted in targeted regions that were selected on the basis of observed sightings of otters by biologists and local residents (Esslinger and Bodkin, 2009). Surveys before 1995 were conducted primarily using small vessels driven parallel to shore while observers onboard counted visible animals (Pitcher, 1989). Subsequent surveys were conducted aerially, using strip transect flight paths directed perpendicular to shore (Bodkin and Udevitz, 1999). Surveys were repeated in 1968, 1975, 1988, 1994 and 2003, with the spatial breadth of the surveys changing based on observations of sea otter occupation.

### Data processing

The original Cameron maps and accompanying documentation were scanned from the archives of the Alaska State Museum. The maps were uploaded into QGIS (QGIS.org, 2021) and georeferenced to match the associated coastline in Bing Satellite imagery using the EPSG:3857 WGS 84/Pseudo-Mercator projection. Kelp beds were digitized both as polygons (as they appear in the Cameron maps) and as maximum linear extent, meaning that they could be matched to the ShoreZone data format more readily. The Cameron surveys recorded metadata for each kelp bed, including canopy species composition (dragon kelp, giant kelp and bull kelp) as a singular species or mixed canopy, with the proportion of area occupied estimated in tenths; and bed density as a six-point scale from very thin to very heavy.

The ShoreZone ESRI shapefiles of canopy kelp in Southeast Alaska (regions 10–12) and Southcentral Alaska (regions 3–8) were downloaded from the ShoreZone website (ShoreZone, 2022), along with accompanying metadata on the identification number (region, area and unit); wave exposure as a six-point scale from very protected to very exposed; and the canopy species composition (dragon kelp, giant kelp and bull kelp) of each bed, including whether a given species was present as patchy or continuous. Patchy was defined as being visible in less than half of the along-shore unit length, and continuous was defined as more than half (Harper and Morris, 2014). Quality assurances and control of biological observations were achieved through the review of 10% of all observations by mappers by another mapper (Cook *et al.*, 2017).

To combine the two datasets, we divided the Cameron kelp beds into 10 m increments and associated each increment with the nearest ShoreZone unit using the *sf* package in R (Pebesma, 2018). For each kelp bed unit in both datasets, we applied a multiplier to account for variation in the thickness of the bed: for the ShoreZone data, if any species was described as continuous for a given bed, then the bed was determined to be continuous and the linear extent multiplied by 0.75; if all species present were listed as patchy, then the bed was determined to be patchy and multiplied by 0.25. For the Cameron data, we multiplied the linear extent of beds by 0.75 if they were described as ‘very heavy’ to ‘medium heavy’ or by 0.25 if they were described as ‘medium’ to ‘very thin’. In order to compare the species composition of beds in the two datasets, we classified a bed as single species, mixed two species or mixed three species, for a total of seven possible combinations.

Given that it was unclear precisely where the Cameron expeditions mapped, analyses were spatially limited to units that contained kelp in the Cameron surveys (Fig. 1).

**Fig. 2.**
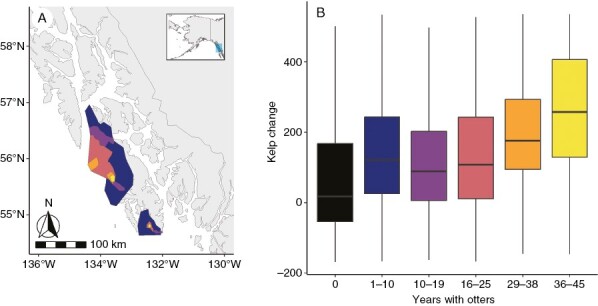
(A) The spatial extent of otters observed in aerial surveys in 1968 (yellow), 1975 (orange), 1988 (pink), 1994 (purple) and 2003 (blue). (B) The influence of otter occupation on the change in kelp between the ShoreZone and Cameron surveys. Kelp change is the linear extent of kelp in the Cameron surveys in 1913 subtracted from the linear extent of kelp in the ShoreZone 2004–2013 surveys on a per unit basis (in metres). ‘Years with otters’ indicates the number of years between the ShoreZone surveys and when otters were first observed in an aerial survey in a given location. The horizontal line of the box plot represents the median, the box represents the first and third quartiles, and the vertical line represents 1.5 times the difference between the first and third quartiles. Outliers are excluded from the graph but were included in the analysis.

We also incorporated data from aerial surveys of sea otter range in southeast Alaska from 1968, 1975, 1988, 1994 and 2003 (Fig. 2A). Initially, we overlapped the Cameron and ShoreZone kelp survey data by the observed spatial extent of the sea otter range for each survey year and assigned a presence value to each unit within the area of overlap. We then combined all the data and summed the number of surveys with observed otters, such that units in areas where otters have been observed since 1968 had a value of five, units in areas with otters observed since 2003 had a value of one, and units in areas where otters were never seen in any of the surveys had a value of zero. Based on the survey polygons, otter ranges only expanded, they did not contract. Therefore, the number of surveys with observed otters is an accurate representation of the length of time otters have been observed in an area (Esslinger and Bodkin, 2009).

### Data analysis

We first asked whether kelp extent per unit differed between the two datasets using Welch’s two-sample *t*-test (Welch, 1947). We then used linear models to ask whether differences in kelp extent between the Cameron and ShoreZone surveys differed across space and across gradients of wave exposure. In all models, the independent variable was the difference between the adjusted linear extent of kelp in the ShoreZone surveys minus the adjusted linear extent of kelp in the Cameron surveys, per unit. This means that, for a given unit, a positive number indicated a longer extent of kelp in the ShoreZone survey. We analysed Southeast and Southcentral Alaska separately. To test for differences across space, we used ShoreZone region as the dependent variable. To test for differences across wave exposure, we converted the ShoreZone wave exposure classifications of ‘very protected’ to ‘very exposed’ to a six-point scale. All statistical analyses were conducted in R (R Core Team, 2022).

Using a similar approach with linear models, we asked whether areas with a longer duration of otter presence have more kelp per unit in ShoreZone compared with the Cameron surveys. We also assessed whether wave exposure changed the pattern by incorporating an exposure interaction into the linear model.

To assess whether the species composition of the canopies changed between the two datasets, we considered change per unit between the two surveys, in addition to the total number of units that contained a given species in each survey. To analyse change, we assigned a zero if the species composition of the canopy did not change (e.g. dragon kelp to dragon kelp) or a one if it did change (e.g. dragon to bull kelp or dragon–bull kelp mixed to only bull kelp). We used a binomial regression with species change (0/1) as the dependent variable and species classification in Cameron survey as the independent variable. This approach allowed us to ask whether certain species became more dominant in the ShoreZone surveys than they were in the historical Cameron surveys.

## RESULTS

### Changes in kelp extent

**Fig. 3.**
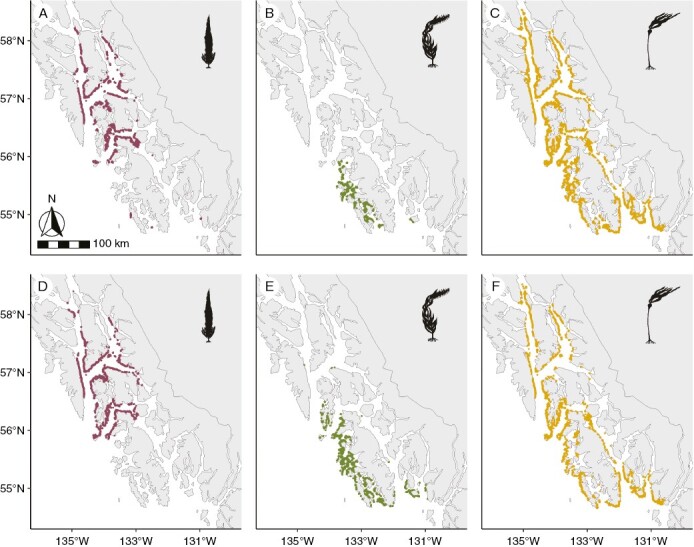
Kelp extent broken up by survey and species in Southeast Alaska. (A–C) Cameron surveys in 1913. (D–F) ShoreZone surveys in 2004–2013. The extent of dragon kelp (*Eularia fistulosa*) is indicated by maroon lines in panels A and D; giant kelp (*Macrocystis pyrifera*) is indicated by green lines in panels B and E; and bull kelp (*Nereocystis luetkeana*) is indicated by gold lines in panels C and F.

**Fig. 4.**
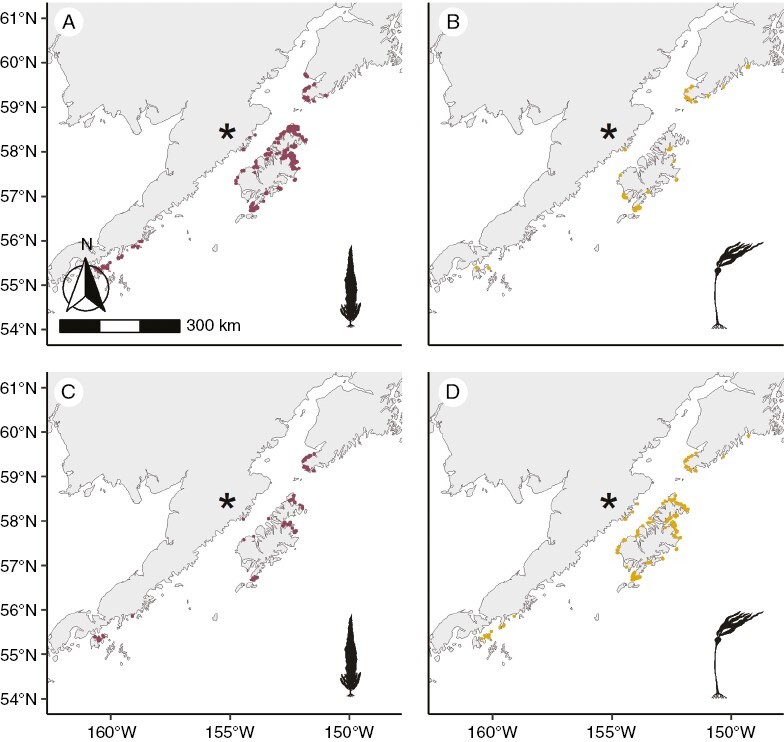
Kelp extent broken up by survey and species in Southcentral Alaska. (A, B) Cameron surveys in 1913. (C, D) ShoreZone surveys in 2004–2013. The extent of dragon kelp (*Eularia fistulosa*) is indicated by maroon lines in panels A and C, and bull kelp (*Nereocystis luetkeana*) is indicated by gold lines in panels B and D. The asterisk indicates the location of the Novarupta volcano.

Overall, 4985 km of kelp were mapped in the study region from the ShoreZone surveys, accounting for adjustments based on whether a bed was patchy or continuous. Of that number, 4567 km were from Southeast Alaska and 418 km from Southcentral Alaska. In the Cameron surveys, 3043 km were mapped, accounting for adjustments based on bed thickness, with 2737 km from Southeast Alaska and 307 km from Southcentral Alaska. The difference between the two datasets on a per-unit basis was significant (Southeast: Cameron mean = 206 m, ShoreZone mean = 345 m, *t* = −31, *P* < 0.001; Southcentral: Cameron mean = 203 m, ShoreZone mean = 277 m, *t* = −5, *P* < 0.001). In Southeast Alaska in both surveys, dragon kelp was encountered mainly in the north, giant kelp along the exposed coast and bull kelp throughout (Fig. 3). In Southcentral Alaska in both surveys, both dragon and bull kelp were distributed throughout, and giant kelp was not observed (Fig. 4).

**Fig. 5.**
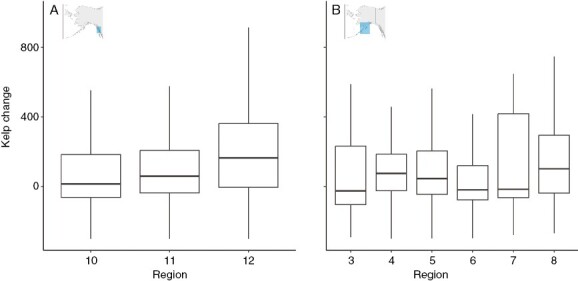
Spatial variability of canopy kelp change in (A) Southeast Alaska and (B) Southcentral Alaska. Kelp change is the linear extent of kelp in the Cameron surveys in 1913 subtracted from the linear extent of kelp in the ShoreZone 2004–2013 surveys on a per unit basis (in metres). Regions are ShoreZone classifications. The horizontal line of the box plot represents the median, the box represents the first and third quartiles, and the vertical line represents 1.5 times the difference between the first and third quartiles. Outliers are excluded from the graph but were included in the analysis.

In Southeast Alaska, the difference in kelp extent per unit differed significantly across regions (Fig. 5A). The smallest difference between the two surveys was observed in the northern region, with slightly more kelp in the ShoreZone surveys compared with the Cameron surveys (region 10; estimate = 69 m, *t* = 9, *P* < 0.001). The central region had a greater difference (region 11; estimate = 94 m, *t* = 3, *P* = 0.00369), and the southern region had the greatest difference between the two surveys (region 12; estimate = 274 m, *t* = 21, *P* < 0.001).

In Southcentral Alaska, kelp likewise differed significantly by region (Fig. 5B). Region 3, which comprises the outer coast of the Kenai Peninsula, had significantly more kelp in the ShoreZone surveys than all other regions except region 8 (estimate = 244 m, *t* = 4, *P* < 0.001). Region 4, representing the Kachemak Bay on the inner coast of the Kenai Peninsula, had more kelp in the Cameron survey (estimate = −88 m, *t* = −5, *P* < 0.001). Region 5, along the outer coast of Kodiak island, region 6, along the coast of Kodiak Island that borders Shelikof Strait, and region 7, along the mainland portion of Shelikof Strait, including Katmai National Park, also differed significantly from region 3 (region 5: estimate = 104 m, *t* = −2, *P* < 0.001; region 6: estimate = 15 m, *t* = −4, *P* = 0.001; region 7: estimate = 7 m, *t* = −3, *P* = 0.005). Region 8 is the beginning of the Aleutian Island chain and did not differ significantly from region 3 (estimate = 149 m, *t* = −1, *P* = 0.15).

**Fig. 6.**
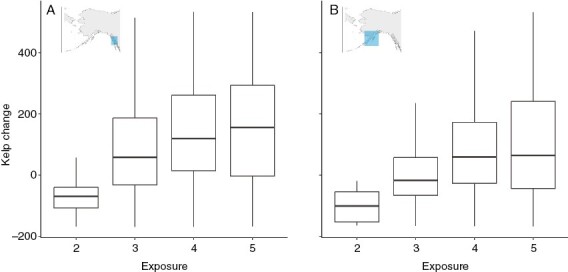
Influence of wave exposure on canopy kelp change in (A) Southeast Alaska and (B) Southcentral Alaska. Kelp change is the linear extent of kelp in the Cameron surveys in 1913 subtracted from the linear extent of kelp in the ShoreZone 2004–2013 surveys on a per unit basis (in metres). Wave exposures were ShoreZone classifications and determined by the amount of fetch in a given unit: 1 indicates ‘very protected’, 2 is ‘protected’, 3 is ‘semi-protected’, 4 is ‘semi-exposed’, 5 is ‘exposed’, and 6 is ‘very exposed’. The horizontal line of the box plot represents the median, the box represents the first and third quartiles, and the vertical line represents 1.5 times the difference between the first and third quartiles. Outliers are excluded from the graph but were included in the analysis.

In both Southeast and Southcentral Alaska, there was significantly more kelp in the ShoreZone surveys compared with the Cameron surveys as wave exposure increased (Fig. 6; Southeast: estimate = 137 m, *t* = 25, *P* < 0.001; Southcentral: estimate = 77, *t* = 4, *P* < 0.001).

### Changes in canopy species composition

**Fig. 7.**
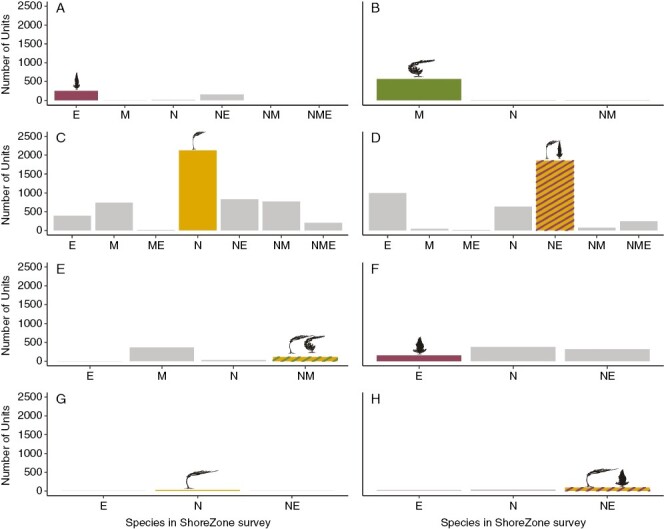
Change in species composition on a per unit basis in Southeast Alaska (A–E) and Southcentral Alaska (F–H). Each panel represents the number of units dominated by a given species in the Cameron surveys (A and F, dragon kelp; B, giant kelp; C and G, bull kelp; D and H, mixed bull–dragon kelp; E, mixed bull–giant kelp), and the bars in each panel show the number of units dominated by each species or mix of species in the ShoreZone surveys (E: dragon kelp; M: giant kelp; N: bull kelp). The numbers of units that had no change in species composition are highlighted in colour and with kelp species icons.

**Fig. 8.**
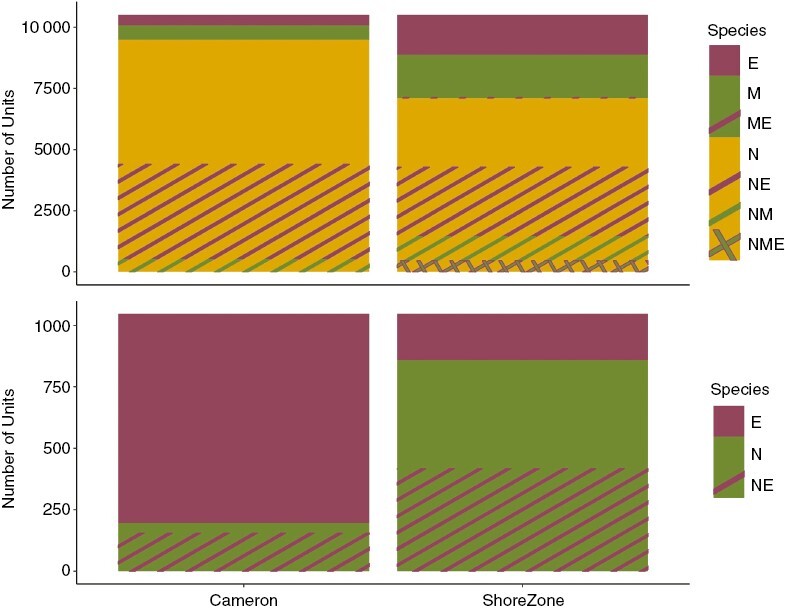
The total number of units of each species or species combination (E: dragon kelp; M: giant kelp; N: bull kelp) by survey and by region (A, Southeast Alaska; B, Southcentral Alaska).

When considering only units that had kelp in both surveys, we observed significant changes in the species composition of the kelp beds in both Southeast and Southcentral Alaska (Figs 7 and 8).

In Southeast Alaska, giant kelp was the most likely to have changed between the two surveys, showing a significant increase in representation (estimate = −2.8, *z* = −12, *P* < 0.001). Mixed bull–giant kelp beds were the next most likely to change, with a significant decrease in representation; these beds were largely replaced by giant kelp (estimate = 1.6, *z* = 11, *P* < 0.001). Bull kelp (estimate = 0.6, *z* = 6.5, *P* < 0.001) and mixed bull–dragon kelp (estimate = 0.4, *z* = 4, *P* < 0.001) both decreased significantly in representation. Finally, dragon kelp showed a small but significant increase in representation between the two surveys (estimate = −0.3, *z* = −3, *P* < 0.001) (Figs 7 and 8).

In Southcentral Alaska, bull kelp was the most likely to have changed between the two surveys (estimate = −1.9, *z*-value = −6, *P* < 0.001), followed by mixed bull–dragon kelp beds (estimate = −1.8, *z*-value = −9, *P* < 0.001), both of which showed an increase in representation. Dragon kelp beds decreased significantly in representation (estimate = 1.5, *z*-value = 17, *P* < 0.001) (Figs 7 and 8).

### Influence of otters on kelp extent

The difference in kelp extent per unit between the ShoreZone and Cameron surveys was greater in areas where otters had been observed for longer (Fig. 2B; estimate = 47 m, *t* = 17, *P* < 0.001), meaning that areas with a longer history of otter occupation also had more kelp in recent surveys than areas with no or only recent otter occupation. The relationship between otter occupation time, change in kelp per unit and exposure was complex, with otter occupation time, exposure and the interaction between the two all being significant predictors of the change in kelp per unit (interaction estimate = −38; *t*-value = −9; *P* < 0.001).

## DISCUSSION

By drawing upon diverse datasets (a previously undigitized shipboard survey of 20th century canopy kelp, modern aerial surveys of 21st century kelp in Alaska, and aerial surveys of sea otter range expansion following initial reintroduction in 1965), we were able to elucidate patterns of change and stability in an important nearshore habitat in a data-scarce region. The results of our analyses suggest remarkable stability in the overall coverage of canopy kelp habitat in Southeast Alaska and an expansion of canopy kelp habitat in Southcentral Alaska. We also observed evidence for the keystone role that sea otters play in kelp ecosystems: areas with longer observations of otter occupation had greater increases in kelp extent. Finally, we found an increase in the canopy dominance of bull and giant kelp relative to dragon kelp. This combination of stability and change highlights the dynamic nature of canopy kelp ecosystems, dominated by annual species in Alaska, and the importance of surveys to be able to catalogue that change.

Our analyses suggest that the linear extent of canopy kelp increased between 1913 and the early 2000s, contrary to general trends of kelp decline observed along much of the Pacific Basin (Krumhansl *et al.*, 2016). Declines along the western North American coast, including Northern California and the Puget Sound, have been especially severe in recent decades and have probably been driven by the combination of warming sea surface temperatures and the reduction of herbivore predators, such as large rockfish, lingcod, sunflower stars and sea otters (Rogers-Bennett and Catton, 2019; Berry *et al.*, 2021; Hollarsmith *et al.*, 2022). However, there are important exceptions to this downward trend, such as in the high-latitude giant kelp ecosystems of Southern Chile (Mora-Soto *et al.*, 2021) and parts of outer Vancouver Island (Starko *et al.*, 2022). In both cases, the authors observed higher persistence of kelp in areas of high wave exposure, the same pattern we observed in our data. Notably, we observed increases in kelp extent even in less exposed areas, despite the fact that the Gulf of Alaska has warmed significantly over the past century (Danielson *et al.*, 2022).

Our observations of kelp increase present the hopeful possibility of high-latitude refugia for these cold-adapted species; however, there are important caveats to consider. Observed increases in kelp extent in Southeast and Southcentral Alaska between the two surveys, especially in areas of high wave exposure, could be exaggerated by differences in how the surveys were conducted. Unlike the boat-based Cameron surveys, the aerial ShoreZone surveys were unencumbered by marine conditions and were able to target low tides consistently, in order to maximize the likelihood of observing canopy kelp (Cook *et al.*, 2017). The Cameron surveys in Southcentral Alaska explicitly state that extreme weather prevented them from conducting full surveys in parts of Kodiak Island and the Alaska Peninsula (Cameron, 1915). Although not specified in the documentation from the Cameron surveys in Southeast Alaska, it would be reasonable to assume that more exposed beds were harder to visit and therefore less likely to be included in the final dataset. Additionally, these surveys do not include the last decade of particularly anomalous conditions (Litzow *et al.*, 2020). The record-breaking Pacific marine heatwave event of 2014–2016 profoundly altered subtidal kelp ecosystems along the west coast of North America, from Baja California, Mexico, to Washington state, USA (Arafeh-Dalmau *et al.*, 2019; Rogers-Bennett and Catton, 2019; Starko *et al.*, 2022; Tolimieri *et al.*, 2023). Intertidal monitoring in Southcentral Alaska suggests that macroalgae species were impacted by the heatwave (Weitzman *et al.*, 2021).

Increases of canopy kelp in Southeast Alaska seem almost entirely attributable to the range expansion of sea otters. Researchers have closely tracked the effects of otters since the reintroduction of 403 individuals between 1965 and 1969 (Esslinger and Bodkin, 2009). The diet of sea otters in the inside waters of Southeast Alaska tends to be dominated by bivalves rather than urchins (Kvitek and Oliver, 1992; LaRoche *et al.*, 2021), and the classic otter-mediated trophic cascade has not been observed in seagrass ecosystems in the region (Raymond *et al.*, 2021), both of which suggested that the effect of otters on kelp ecosystems might be less pronounced than in other regions (e.g. Estes and Palmisano, 1974). However, we found that areas with a longer history of otter occupation had a much greater spatial extent of kelp in recent compared with historical surveys, with very little change between the two surveys in areas outside the sea otter range. These findings suggest that as otters continue to expand their range in Southeast Alaska, kelp ecosystems will be likely also to expand, barring other climatic or trophic disturbances.

The increases in kelp extent in Southcentral Alaska between the two surveys can probably be attributed entirely to recovery of the ecosystem following the massive and catastrophic 1912 eruption of the Novarupta volcano, previously thought to be Mount Katmai. This was the largest volcanic eruption of the 20th century, and observers who visited the region shortly after the eruption found that ‘marine life was affected to a larger degree than perhaps would be expected [ … ]. Kelp is apparently dead as far as the eastern end of Afognak Island’ (Martin, 1913; Hildreth and Fierstein, 2012). The Cameron expedition likewise attributed the lack of kelp in Shelikof Strait (ShoreZone regions 6 and 7) to the effects of the eruption, which happened only 1 year before the surveys and resulted in ≤50 cm of ash falling in that region (Cameron, 1915; Fierstein and Hildreth, 1992). Thus, although our findings from Southcentral Alaska are not necessarily indicative of persistence in the face of a changing climate, they do illustrate the potential of kelp ecosystems to recover from extreme point disturbances. Surveys of kelp recovery after large disturbances, such as volcanic eruptions or tsunamis, have found returns of canopy kelp abundance to pre-disturbance levels within years (e.g. Walker *et al.*, 2013; Muraoka *et al.*, 2017), although Thomsen *et al.* (2021) observed persistent extirpation of large brown macroalgae (*Durvillaea antarctica* and *Durvillaea willana*) 4 years after a major earthquake in New Zealand.

We also found evidence that the species composition of the beds might have changed in the past century, with more thermally tolerant species (giant kelp in Southeast Alaska and bull kelp in Southcentral Alaska) increasing more than the more cold-adapted species (dragon kelp). In Southeast Alaska, this expansion of giant kelp, in particular, could be the consequence of climate-driven competitive advantage of this thermally tolerant species (Muth *et al.*, 2019; Hollarsmith *et al.*, 2020). Climate-driven shifts in dominance have been observed in other macroalgal ecosystems (Straub *et al.*, 2016). In Southcentral Alaska, our interpretation of species dominance must again account for the effects of the volcano, because the altered water quality might have affected each species differentially.

It is also possible the patterns of species canopy cover are a relic of differences in survey methodology, in that the Cameron surveys were conducted all in one season, such that the first site (Ketchikan) was visited in early spring and the last site (unspecified) in early autumn, whereas the ShoreZone surveys took place during spring and summer across multiple years. In Southeast Alaska today, giant kelp generally reaches peak biomass in June (Bell and Kroeker, 2022) and bull kelp in late July (T. Peeples, University of Alaska Fairbanks, Juneau, pers. comm.). We were unable to find information on dragon kelp. The Cameron survey observed peak biomass of bull kelp in August and dragon kelp in late July (Cameron, 1915). Therefore, species observations could be confounded by differences in the timing of when a given bed was surveyed. This is a system dominated by annual species, meaning that the dominant species of a given bed might also switch on annual and interannual scales depending on oceanographic or ecological conditions (Grubb, 1988; Bell *et al.*, 2020).

In order to detect future changes in kelp canopy abundance and species composition better in this vast and remote region, we propose the establishment of sentinel kelp beds that can be monitored at regular intervals using remote sensing techniques (e.g. Cavanaugh *et al.*, 2021) and through collaboration and engagement with coastal communities (e.g. Diggon *et al.*, 2022). Potential monitoring locations could include currently known range limits, such as Frederick Sound for dragon kelp (Fig. 1, boxes 11.04 and 11.05) or areas of species expansion, such as Kuiu Island for giant kelp (Fig. 1, box 11.06) or Shelikof Strait for bull kelp (Fig. 1, boxes 6.01–6.03). Sites could also prioritize proximity to villages and towns for ease of access for community-science initiatives, or areas of particular cultural or economic importance, such as traditional or commercial harvesting grounds. Novel tools, such as KelpWatch (Bell *et al.*, 2023), which uses Landsat satellite imagery to detect kelp, can also be used to assess change in remote areas where physical access is challenging. Although satellite imagery can be a powerful tool for mapping kelp (e.g. Bell *et al*., 2018), the high currents, high tidal flux and high cloud cover common to coastal Alaska make satellite imagery less reliable for mapping the fringing kelp beds in this glacially carved region (Stekoll *et al.*, 2006; Cavanaugh *et al.*, 2021). Regardless of the approach adopted, it will be crucial to monitor this expansive and important habitat at a higher temporal resolution than 100 years, especially as the environment continues to change rapidly.

## References

[CIT0058] Arafeh-Dalmau N , Montaño-Moctezuma G, Martínez JA, Beas-Luna R, Schoeman DS, Torres-Moye G. 2019. Extreme marine heatwaves alter kelp forest community near its equatorward distribution limit. Frontiers in Marine Science 6: 499. doi:10.3389/fmars.2019.00499.

[CIT0061] Bell TW , Allen JG, Cavanaugh KC, Siegel DA. 2018. Three decades of variability in California’s giant kelp forests from the Landsat satellites. Remote Sensing of Environment 238: 110811. doi:10.1016/j.rse.2018.06.039. https://linkinghub.elsevier.com/retrieve/pii/S0034425718303171 (accessed 14 August 2018, date last accessed).

[CIT0001] Bell TW , Allen JG, Cavanaugh KC, Siegel DA. 2020. Three decades of variability in California’s giant kelp forests from the Landsat satellites. Remote Sensing of Environment 238: 110811. doi:10.1016/j.rse.2018.06.039.

[CIT0002] Bell TW , Cavanaugh KC, Saccomanno VR, et al. 2023. Kelpwatch: a new visualization and analysis tool to explore kelp canopy dynamics reveals variable response to and recovery from marine heatwaves. PLoS One 18: e0271477. doi:10.1371/journal.pone.0271477. 36952444 PMC10035835

[CIT0060] Bell LE , Kroeker KJ. 2022. Standing Crop, Turnover, and Production Dynamics of Macrocystis pyrifera and Understory Species Hedophyllum nigripes and Neoagarum fimbriatum in High Latitude Giant Kelp Forests. Journal of Phycology 58: 773–788. 36302142 10.1111/jpy.13291 PMC10100489

[CIT0003] Berry HD , Mumford TF, Christiaen B, et al. 2021. Long-term changes in kelp forests in an inner basin of the Salish Sea. PLoS One 16: e0229703. doi:10.1371/journal.pone.0229703. 33596204 PMC7888675

[CIT0004] Bodkin JL , Udevitz MS. 1999. An aerial survey method to estimate sea otter abundance. In: Laake JL, Robertson DG, Amstrup SC, Manly BFJ, eds. Marine mammal survey and assessment methods. London: CRC Press, 13–26. doi:10.1201/9781003211167.

[CIT0005] Bolton JJ. 2010. The biogeography of kelps (Laminariales, Phaeophyceae): a global analysis with new insights from recent advances in molecular phylogenetics. Helgoland Marine Research 64: 263–279. doi:10.1007/s10152-010-0211-6.

[CIT0006] Breen PA , Carson TA, Foster JB, Stewart EA. 1982. Changes in subtidal community structure associated with British Columbia sea otter transplants. Marine Ecology Progress Series 7: 13–20. doi:10.3354/meps007013.

[CIT0007] Cameron F. 1915. Potash from kelp. United States Department of Agriculture, Office of the Secretary. Report No. 100.

[CIT0008] Cavanaugh KC , Bell T, Costa M, et al. 2021. A review of the opportunities and challenges for using remote sensing for management of surface-canopy forming kelps. Frontiers in Marine Science 8: 1–15. doi:10.3389/fmars.2021.753531.35685121

[CIT0009] Cook S , Daley S, Morrow K, Ward S. 2017. ShoreZone coastal imaging and habitat mapping protocol. Prepared for NOAA, National Marine Fisheries Service, Alaska Region, Habitat Conservation Division, Juneau, AK, USA by Coastal and Ocean Resources.

[CIT0010] Costa M , Le Baron N, Tenhunen K, et al. 2020. Historical distribution of kelp forests on the coast of British Columbia: 1858–1956. Applied Geography 120: 102230. doi:10.1016/j.apgeog.2020.102230.

[CIT0011] Danielson SL , Hennon TD, Monson DH, et al. 2022. Temperature variations in the northern Gulf of Alaska across synoptic to century-long time scales. Deep Sea Research Part II: Topical Studies in Oceanography 203: 105155. doi:10.1016/j.dsr2.2022.105155.

[CIT0012] Diggon S , Bones J, Short CJ, et al. 2022. The marine plan partnership for the North Pacific Coast – MaPP: a collaborative and co-led marine planning process in British Columbia. Marine Policy 142: 104065. doi:10.1016/j.marpol.2020.104065.

[CIT0013] Eger AM , Marzinelli EM, Beas-Luna R, et al. 2023. The value of ecosystem services in global marine kelp forests. Nature Communications 14: 1894. doi:10.1038/s41467-023-37385-0. PMC10113392 37072389

[CIT0014] Erlandson JM , Graham MH, Bourque BJ, Corbett D, Estes JA, Steneck RS. 2007. The kelp highway hypothesis: marine ecology, the coastal migration theory, and the peopling of the Americas. The Journal of Island and Coastal Archaeology 2: 161–174. doi:10.1080/15564890701628612.

[CIT0015] Esslinger GG , Bodkin JL. 2009. Status and trends of sea otter populations in Southeast Alaska, 1969–2003: U.S. Geological Survey Scientific Investigations Report 2009-5045, 18 pp.

[CIT0016] Estes JA , Duggins DO. 1995. Sea otters and kelp forests in Alaska: generality and variation in a community ecological paradigm. Ecological Monographs 65: 75–100. doi:10.2307/2937159.

[CIT0017] Estes JA , Palmisano JF. 1974. Sea otters: their role in structuring nearshore communities. Science 185: 1058–1060. doi:10.1126/science.185.4156.1058. 17738247

[CIT0018] Fierstein J , Hildreth W. 1992. The plinian eruptions of 1912 at Novarupta, Katmai National Park, Alaska. Bulletin of Volcanology 54: 646–684. doi:10.1007/bf00430778.

[CIT0019] Grubb PJ. 1988. The uncoupling of disturbance and recruitment, two kinds of seed bank, and persistence of plant populations at the regional and local scales. Annales Zoologici Fennici 25: 23–36.

[CIT0020] Harper JR , Morris MC. 2014. ShoreZone coastal imaging and habitat mapping protocol. Prepared for Bureau of Ocean Energy Management for contract M11PC0037 by Nuka Research and Planning Group, LLC.

[CIT0021] Hildreth W , Fierstein J. 2012. The Novarupta-Katmai eruption of 1912—largest eruption of the twentieth century; centennial perspectives: U.S. Geological Survey Professional Paper 1791, 259 pp. https://pubs.usgs.gov/pp/1791/

[CIT0022] Hollarsmith JA , Buschmann AH, Camus C, Grosholz ED. 2020. Varying reproductive success under ocean warming and acidification across giant kelp (*Macrocystis pyrifera*) populations. Journal of Experimental Marine Biology and Ecology 522: 151247. doi:10.1016/j.jembe.2019.151247.

[CIT0023] Hollarsmith JA , Andrews K, Naar N, et al. 2022. Toward a conceptual framework for managing and conserving marine habitats: a case study of kelp forests in the Salish Sea. Ecology and Evolution 12: e8510. doi:10.1002/ece3.8510. 35136559 PMC8809449

[CIT0024] Konar B. 2000. Limited effects of a keystone species: trends of sea otters and kelp forests at the Semichi Islands, Alaska. Marine Ecology Progress Series 199: 271–280. doi:10.3354/meps199271.

[CIT0025] Krumhansl KA , Okamoto DK, Rassweiler A, et al. 2016. Global patterns of kelp forest change over the past half-century. Proceedings of the National Academy of Sciences of the United States of America 113: 13785–13790. doi:10.1073/pnas.1606102113. 27849580 PMC5137772

[CIT0026] Kvitek R , Oliver J. 1992. The influence of sea otters on prey communities in southeast Alaska. Marine Ecology Progress Series 82: 103–113. doi:10.3354/meps082103.

[CIT0027] LaRoche N , King S, Rogers M, Eckert G, Pearson H. 2021. Behavioral observations and stable isotopes reveal high individual variation and little seasonal variation in sea otter diets in Southeast Alaska. Marine Ecology Progress Series 677: 219–232. doi:10.3354/meps13871.

[CIT0028] Lindeberg MR , Lindstrom SC. 2016. Field guide to seaweeds of Alaska. Fairbanks: Alaska Sea Grant. ISBN: 978-1-56612-156-9.

[CIT0029] Litzow MA , Hunsicker ME, Ward EJ, et al. 2020. Evaluating ecosystem change as Gulf of Alaska temperature exceeds the limits of preindustrial variability. Progress in Oceanography 186: 102393. doi:10.1016/j.pocean.2020.102393.

[CIT0030] Marshall KN , Duffy-Anderson JT, Ward EJ, Anderson SC, Hunsicker ME, Williams BC. 2019. Long-term trends in ichthyoplankton assemblage structure, biodiversity, and synchrony in the Gulf of Alaska and their relationships to climate. Progress in Oceanography 170: 134–145. doi:10.1016/j.pocean.2018.11.002.

[CIT0031] Martin GC. 1913. The recent eruption of Katmai Volcano in Alaska: an account of one of the most tremendous volcanic explosions known in history. National Geographic Magazine 24: 131–181.

[CIT0032] McPherson ML , Finger DJI, Houskeeper HF, et al. 2021. Large-scale shift in the structure of a kelp forest ecosystem co-occurs with an epizootic and marine heatwave. Communications Biology 4: 298. doi:10.1038/s42003-021-01827-6. 33674760 PMC7935997

[CIT0033] Mora-Soto A , et al. 2021. One of the least disturbed marine coastal ecosystems on Earth: spatial and temporal persistence of Darwin’s sub-Antarctic giant kelp forests. Journal of Biogeography 48: 2562–2577.

[CIT0034] Muraoka D , Tamaki H, Takami H, Kurita Y, Kawamura T. 2017. Effects of the 2011 Great East Japan Earthquake and tsunami on two kelp bed communities on the Sanriku coast. Fisheries Oceanography 26: 128–140. doi:10.1111/fog.12198.

[CIT0035] Muth AF , Graham MH, Lane CE, Harley CDG. 2019. Recruitment tolerance to increased temperature present across multiple kelp clades. Ecology 100: e02594. doi:10.1002/ecy.2594. 30615200

[CIT0036] Naar N. 2020. Appendix B- the cultural importance of kelp for Pacific northwest tribes. In: Calloway M, Oster D, Berry H, Mumford T, Naar N, Peabody B, Hart L, Tonnes D, Copps S, Selleck J, Allen B, Toft J. eds. Puget Sound kelp conservation and recovery plan. Prepared for NOAA-NMFS, Seattle, WA. 52 pp plus appendices. https://nwstraits.org/media/2957/appendix_b_the-cultural-importance-of-kelp-for-pacific-northwest-tribes.pdf.

[CIT0037] Parnell PE , Miller EF, Cody CEL, Dayton PK, Carter ML, Stebbinsd TD. 2010. The response of giant kelp (*Macrocystis pyrifera*) in southern California to low-frequency climate forcing. Limnology and Oceanography 55: 2686–2702. doi:10.4319/lo.2010.55.6.2686.

[CIT0038] Pauly D , Palomares M-L. 2005. Fishing down marine food web: it is far more pervasive than we thought. Bulletin of Marine Science 76: 15.

[CIT0039] Pebesma E. 2018. Simple features for R: standardized support for spatial vector data. The R Journal 10: 439–446. doi:10.32614/rj-2018-009.

[CIT0040] Pitcher KW. 1989. Studies of Southeastern Alaska sea otter populations: distribution, abundance, structure, range expansion and potential conflicts with shellfisheries. Final Report, Alaska Department of Fish and Game, Cooperative Agreement 14‐16‐0009‐954. U.S. Fish and Wildlife Service, Anchorage, AK, USA.

[CIT0041] QGIS.org. 2021. QGIS geographic information system. QGIS Association. http://www.qgis.org/

[CIT0042] R Core Team. 2022. R: a language and environment for statistical computing. Vienna: R Foundation for Statistical Computing. https://www.R-project.org/

[CIT0043] Raymond WW , Hughes BB, Stephens TA, Mattson CR, Bolwerk AT, Eckert GL. 2021. Testing the generality of sea otter-mediated trophic cascades in seagrass meadows. Oikos 130: 725–738. doi:10.1111/oik.07681.

[CIT0044] Robinson D , Howell D, Sandberg E, Brooks L. 2020. Alaska population overview: 2019 estimates. Juneau: Alaska Department of Labor and Workforce Development. ISSN: 1063-3790.

[CIT0045] Rogers-Bennett L , Catton CA. 2019. Marine heat wave and multiple stressors tip bull kelp forest to sea urchin barrens. Scientific Reports 9: 15050. doi:10.1038/s41598-019-51114-y. 31636286 PMC6803666

[CIT0046] ShoreZone. 2022. Alaska ShoreZone Mapping Website. https://alaskafisheries.noaa.gov/mapping/sz/ (27 April 2022, date last accessed).

[CIT0047] Starko S , Neufeld CJ, Gendall L, et al. 2022. Microclimate predicts kelp forest extinction in the face of direct and indirect marine heatwave effects. Ecological Applications : A Publication of the Ecological Society of America 32: e2673. doi:10.1002/eap.2673. 35584048 PMC13425692

[CIT0048] Stekoll MS , Deysher LE, Hess M. 2006. A remote sensing approach to estimating harvestable kelp biomass. Journal of Applied Phycology 18: 323–334.

[CIT0049] Straub SC , Thomsen MS, Wernberg T. 2016. The dynamic biogeography of the Anthropocene: the speed of recent range shifts in seaweeds. In: Hu Z-M, Fraser C, eds. Seaweed phylogeography: adaptation and evolution of seaweeds under environmental change. Dordrecht: Springer Netherlands, 63–93.

[CIT0050] Teagle H , Hawkins SJ, Moore PJ, Smale DA. 2017. The role of kelp species as biogenic habitat formers in coastal marine ecosystems. Journal of Experimental Marine Biology and Ecology 492: 81–98. doi:10.1016/j.jembe.2017.01.017.

[CIT0051] Thomsen MS , Mondardini L, Thoral F, et al. 2021. Cascading impacts of earthquakes and extreme heatwaves have destroyed populations of an iconic marine foundation species. Diversity and Distributions 27: 2369–2383. doi:10.1111/ddi.13407.

[CIT0052] Thurstan RH , Brittain Z, Jones DS, Cameron E, Dearnaley J, Bellgrove A. 2018. Aboriginal uses of seaweeds in temperate Australia: an archival assessment. Journal of Applied Phycology 30: 1821–1832. doi:10.1007/s10811-017-1384-z.

[CIT0059] Tolimieri N , Shelton AO, Samhouri JF, et al. 2023. Changes in kelp forest communities off Washington, USA, during and after the 2014-2016 marine heatwave and sea star wasting syndrome. Marine Ecology Progress Series 703:47–66.

[CIT0053] Walker LR , Sikes DS, DeGange AR, et al. 2013. Biological legacies: direct early ecosystem recovery and food web reorganization after a volcanic eruption in Alaska. Écoscience 20: 240–251. doi:10.2980/20-3-3603. Taylor & Francis.

[CIT0054] Watanabe J , Harrold C. 1991. Destructive grazing by sea urchins *Strongylocentrotus* spp. in a central California kelp forest: potential roles of recruitment, depth, and predation. Marine Ecology Progress Series 71: 125–141. doi:10.3354/meps071125.

[CIT0055] Weitzman B , Konar B, Iken K, et al. 2021. Changes in rocky intertidal community structure during a marine heatwave in the Northern Gulf of Alaska. Frontiers in Marine Science 8: 1–18. doi:10.3389/fmars.2021.556820.35685121

[CIT0056] Welch BL. 1947. The generalization of ‘Student’s’ problem when several different population variances are involved. Biometrika 34: 28–35. doi:10.1093/biomet/34.1-2.28. 20287819

[CIT0057] Wernberg T. 2021. Marine heatwave drives collapse of kelp forests in Western Australia. In: Canadell JG, Jackson RB, eds. Ecosystem collapse and climate change. *Ecological Studies*, Vol. 241. Cham: Springer, 325–343. doi:10.1007/978-3-030-71330-0_12.

